# Surface-Wetting Characteristics of DLP-Based 3D Printing Outcomes under Various Printing Conditions for Microfluidic Device Fabrication

**DOI:** 10.3390/mi15010061

**Published:** 2023-12-28

**Authors:** Jeon-Woong Kang, Jinpyo Jeon, Jun-Young Lee, Jun-Hyeong Jeon, Jiwoo Hong

**Affiliations:** School of Mechanical Engineering, Soongsil University, 369 Sangdo-ro, Dongjak-gu, Seoul 06978, Republic of Korea; kangjw159@gmail.com (J.-W.K.); junjynpyo@gmail.com (J.J.); ktino27@gmail.com (J.-Y.L.); bread951030@gmail.com (J.-H.J.)

**Keywords:** three-dimensional printing technology, digital light processing, microfluidics, contact angle, contact angle hysteresis

## Abstract

In recent times, the utilization of three-dimensional (3D) printing technology, particularly a variant using digital light processing (DLP), has gained increasing fascination in the realm of microfluidic research because it has proven advantageous and expedient for constructing microscale 3D structures. The surface wetting characteristics (e.g., contact angle and contact angle hysteresis) of 3D-printed microstructures are crucial factors influencing the operational effectiveness of 3D-printed microfluidic devices. Therefore, this study systematically examines the surface wetting characteristics of DLP-based 3D printing objects, focusing on various printing conditions such as lamination (or layer) thickness and direction. We preferentially examine the impact of lamination thickness on the surface roughness of 3D-printed structures through a quantitative assessment using a confocal laser scanning microscope. The influence of lamination thicknesses and lamination direction on the contact angle and contact angle hysteresis of both aqueous and oil droplets on the surfaces of 3D-printed outputs is then quantified. Finally, the performance of a DLP 3D-printed microfluidic device under various printing conditions is assessed. Current research indicates a connection between printing parameters, surface roughness, wetting properties, and capillary movement in 3D-printed microchannels. This correlation will greatly aid in the progress of microfluidic devices produced using DLP-based 3D printing technology.

## 1. Introduction

The advent of three-dimensional (3D) printing technology, which originated from the process of building up three-dimensional structures layer by layer with computer-aided design (CAD) drawings, has had a notable and beneficial influence on various aspects of everyday life [1,2] and in several industrial sectors, such as aerospace [3], automotive [4], and medical applications [5,6]. This can be attributed to its remarkable capacity for producing sophisticated structures, speedy development, and mass customization in contrast to conventional manufacturing methods [1,2].

There has recently been a significant increase in interest regarding the implications of 3D printing technology for the fabrication of microfluidic systems in place of traditional lithography methods, primarily using poly(dimethylsiloxane) (PDMS) [7,8]. This is because 3D printing allows automated, assembly-free 3D fabrication, offering rapid cost reduction as well as rapidly increasing resolution and throughput [7]. Several 3D printing techniques have been used in the field of microfluidics and its applications, including fused deposition modeling (FDM) [9], binder jet 3D printing [10], digital light processing (DLP) [11,12,13], stereolithography (SLA) [14,15], and selective laser sintering [16]. In particular, photopolymerization-based 3D printing technologies, such as SLA and DLP, have shown better print accuracy and print quality when printing chips and making 3D microchannels, even those with complex structures [17]. SLA and DLP are 3D printing techniques that utilize a laser beam (or UV light) and a digital light projector, respectively, to progressively expose liquid photosensitive material, causing it to gradually solidify into the desired object [17].

To create microfluidic systems with these 3D printing technologies and achieve the desired performance, fundamental research on the mechanical and physicochemical properties of photopolymerization-based printed products, based on the printing conditions, remains essential [18,19]. Several researchers have focused on the relationship between printing conditions and the mechanical properties of 3D-printed products in different printing methods [20,21,22,23,24,25]. For instance, Favero et al. [20] evaluated the effect of layer height on accuracy when a model was created with a 3D printer, based on an SLA scheme. Zhang et al. [21] examined the accuracies of DLP and SLA printers at various layer thicknesses and discovered the optimum layer thickness for these printing techniques. Liu et al. [22] explored the impact of printing-layer thickness on mechanical properties and optimized printing conditions through its modulation via fused deposition modeling-based 3D printing. For SLA-manufactured products, Saini et al. [23] investigated the effect of layer directions on mechanical properties, such as tensile, compression, flexural, impact, and fatigue characteristics. Ouassil et al. [24] studied the effect of printing speed on the porosity and tensile characteristics of fused filament 3D-printed materials. Jiang et al. [25] investigated how layer thickness affected the mechanical characteristics and molding accuracy of 3D-printed samples using a DLP scheme. The majority of these prior studies, however, have concentrated on the effects of printing parameters on the mechanical characteristics of 3D-printed objects. 

In microfluidic systems, e.g., when the system size is reduced to the millimeter or micrometer scale, continuous or discontinuous flow (e.g., droplets) is primarily influenced by interfacial tension (or surface tension) rather than volumetric forces such as inertia and gravity [26,27]. Wettability characteristics, such as the contact angle and contact angle hysteresis (CAH, the difference between the advancing and receding contact angles), are widely recognized as crucial physical factors for understanding wetting and the capillary phenomena resulting from interfacial tension [27]. For instance, the physicochemical inhomogeneity of a solid surface causes CAH, which hinders the movement of discontinuous fluids [28,29], like a raindrop clinging to a window. Therefore, a thorough investigation of the wetting properties of 3D-printed objects as a function of printing conditions is essential and crucial to achieving the desired functionality of 3D-printed microfluidic systems.

In the present study, we methodically investigate the surface-wetting characteristics of DLP-based 3D printing outcomes under varied printing conditions to offer crucial and practical guidelines for fabricating microfluidic devices. We primarily examine the correlation between surface roughness and lamination thickness through microscopic and confocal microscopic image analysis. The variations in CA and CAH seen in aqueous and oily droplets are then investigated as a function of lamination direction and thickness. Finally, we analyze the dynamics of liquid flow when driven by capillary action inside simple microchannels that have been fabricated under different printing conditions to evaluate the effect of printing conditions on the functionality of a DLP 3D-printed microfluidic device.

## 2. Materials and Methods

**Figure 1:**
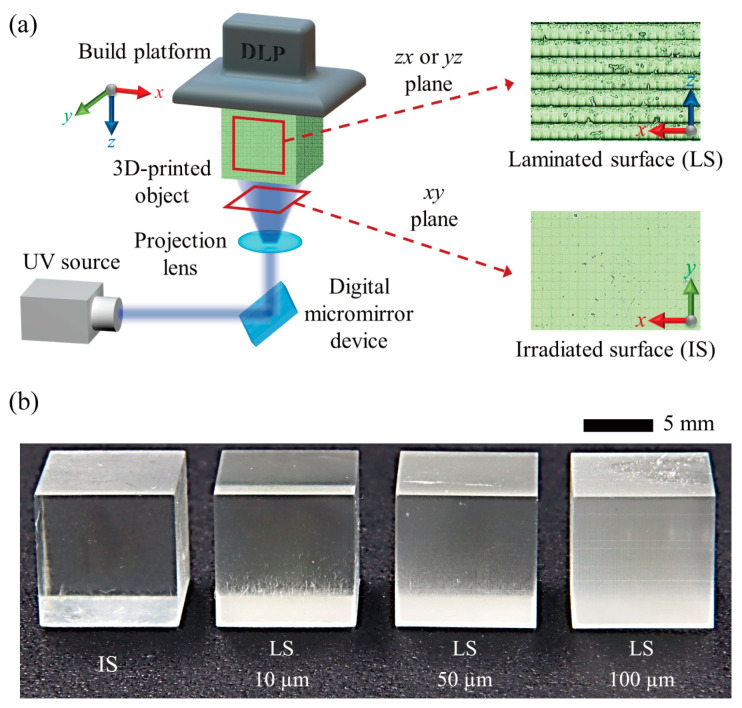
(**a**) Schematic of the DLP-based 3D printing process. (**b**) Actual photographs of the 3D-printed objects under different printing conditions. Here, IS and LS stand for irradiated and laminated surfaces, respectively, while the numerical values indicate the lamination thicknesses.

To examine the effects of 3D printing conditions on the surface characteristics of products, we initially designed a cube of 10 mm and exported the design in the form of STL files using SolidWorks 2020, a professional 3D CAD program (Dassault Systèmes SolidWorks Corp., Waltham, MA, USA). Using Asiga Composer 1.3 (Asiga, Sydney, Australia), the design was segmented into layers and lamination conditions were set for layer thicknesses of 10 μm, 50 μm, and 100 μm. Cubic objects with different layer thicknesses were then manufactured utilizing a DLP-based 3D printer (Asiga MAX X27, Asiga, Australia) and printable resin (PlasClear V2, Asiga, Australia) (Figure 1). This printable resin is a type of UV-curable resin from the diurethane dimethacrylate family. It is widely used in microfluidics due to its many advantageous properties, such as transparency and chemical stability against organic solvents [30,31]. The printing durations for layer thicknesses of 10 μm, 50 μm, and 100 μm were approximately 370, 84, and 51 min, respectively. The same objects were printed in a vertical direction to examine the impact of the lamination direction. To eliminate any remaining photocurable resin, we cleaned the printed products utilizing an ultrasonic cleaner (CPX8800H-E, Branson, MO, USA) and isopropyl alcohol. Subsequently, the printed products were post-cured through the application of UV light with a UV curing apparatus (Flash, Asiga, Australia) to diminish deformation and augment rigidity.

To qualitatively assess the surface roughness of 3D-printed objects created under various printing conditions, we captured microscopic images using an optical microscope (Eclipse Ci-L, Nikon, Tokyo, Japan) and a CCD camera (Fastcam mini UX100, Photron, Tokyo, Japan). A quantitative investigation of surface roughness as a function of printing conditions was also conducted using a confocal laser scanning microscope (LEXT OLS5000, Olympus, Tokyo, Japan) to enable the expected surface roughness and surface wetting characteristics to be correlated. 

Aqueous and oil-liquid phases were prepared to assess the CA of droplets resting on the surfaces of 3D-printed objects created under varied printing conditions. Aqueous liquid phases included deionized (DI) water and a mixture of DI and Tween 20 (1 mM), while oil–liquid phases included mineral oil (Sigma-Aldrich, St. Louis, MO, USA), silicone oil with a viscosity of 50 cSt (Shinetsu, Tokyo, Japan), and hexadecane (Alfa Aesar, Haverhill, MA, USA). Here, Tween 20, otherwise known as polyoxyethylene (20) sorbitan monolaurate, is a water-soluble surfactant belonging to the polysorbate family [32]. It possesses the capability to modify the interaction between solids and liquids by reducing the surface tension of the liquid [33,34]. The surfactant was utilized in experiments to examine the effect of printing conditions on liquid flow in microchannels and to augment capillary action through the reduction of liquid surface tension. To improve the visibility of the aqueous droplets, a small amount (0.2 wt%) of a blue water-soluble dye was added to the aqueous liquid phase. Table 1 shows a summary of the physical properties of the aqueous and oil–liquid phases at room temperature. The viscosities and densities of the liquids were measured using a rotating viscometer (ViscoQC 300 L, Anton Paar, Graz, Austria) and an analytical balance (ME204T, Mettler Toledo, Columbus, OH, USA), respectively. Surface tension was also measured using the image processing of pendant droplets via the public-domain software ImageJ 1.53 (NIH Image, Bethesda, MD, USA). A micropipette was used to carefully dispense tiny aqueous and oil droplets with a volume of 5 μL onto the surfaces of 3D-printed objects, after which their images were captured using a DSLR camera (EOS 90D, Canon, Tokyo, Japan) equipped with a macro lens (MP-E 65 mm, Canon, Japan). The CA of the sessile droplets was measured from the acquired images using a low-bond axisymmetric drop shape analysis (LBADSA) approach via ImageJ software [35]. The LBADSA approach is based on fitting the Young–Laplace equation according to photographic images of axisymmetric sessile drops using a first-order perturbation method. This method is widely recognized for its ability to accurately measure the contact angle of a spherically shaped sessile droplet under low bond number conditions (using the ratio of the surface tension force to the gravitational force). It is freely available and is implemented as a plugin for the open-source program ImageJ [36].

**Figure 2:**
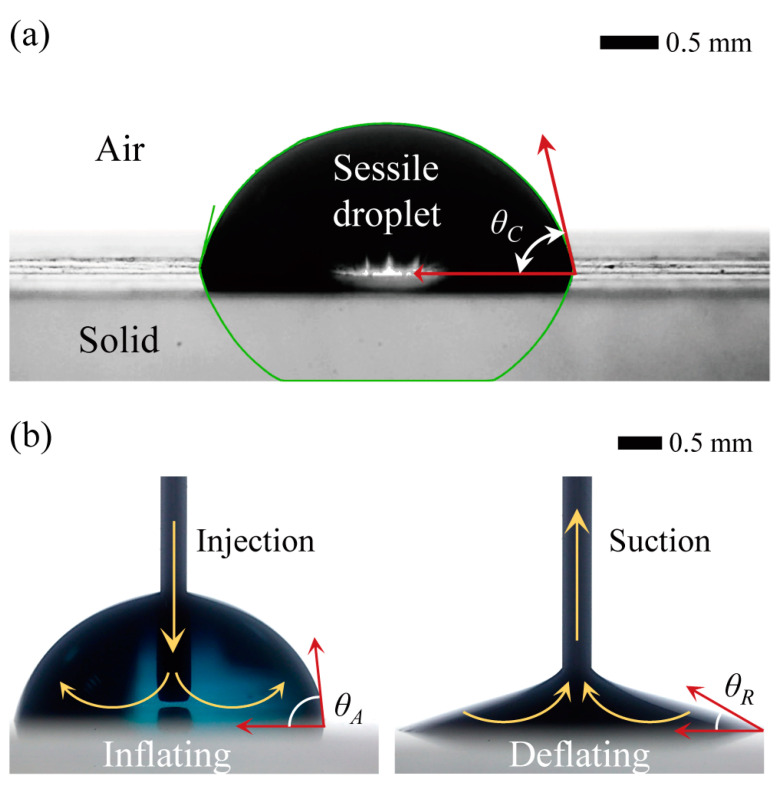
Measurements of: (**a**) the contact angle of a sessile droplet, using a low-bond axisymmetric drop shape analysis approach in ImageJ software; (**b**) changing the advancing and receding contact angles by inflating and deflating the droplet volume, respectively.

To determine the CAH of droplets resting on the surfaces of 3D-printed objects, which is the difference between the advancing (ACA, *θ_A_*) and receding contact angles (RCA, *θ_R_*), we measured the ACA and RCA by inflating and deflating the droplet volumes, respectively (Figure 2). The ACA refers to the maximum CA just before the contact line moves forward on the surface as the droplet volume increases, while the RCA refers to the minimum CA just before the contact line moves backward on the surface as the droplet volume decreases. The ACA and RCA of the sessile droplets can be measured using the LBADSA approach via ImageJ software, which is similar to the CA measurement process. 

**Figure 3:**
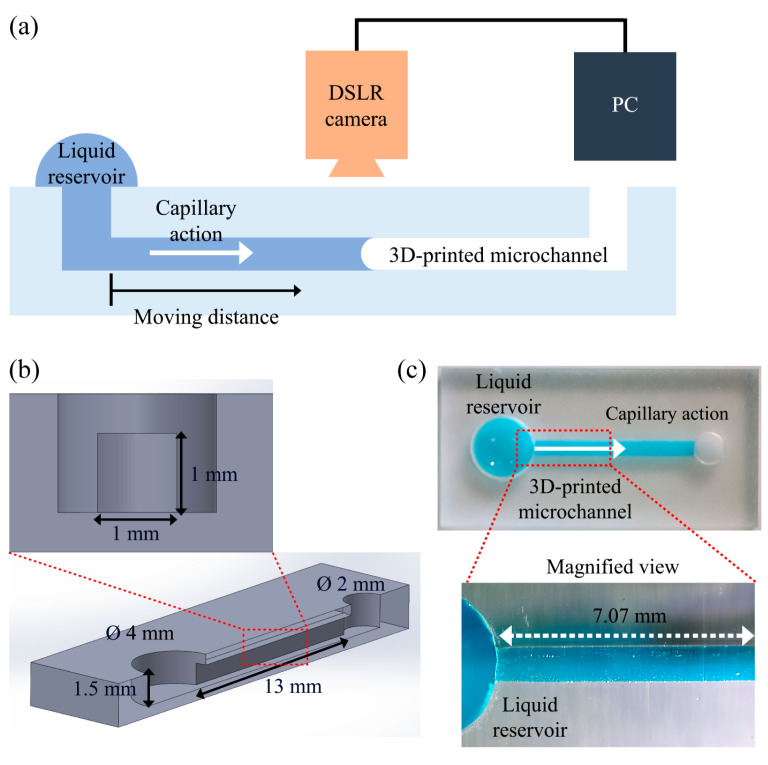
Simple capillary-driven microfluidic device used for evaluating the effect of printing conditions on the performance of DLP 3D-printing: (**a**) schematic, (**b**) drawing, and (**c**) actual photograph.

To evaluate the effect of printing conditions on the functionality of a DLP 3D-printed microfluidic device, we designed a simple microfluidic device that enables the observation of the dynamics of liquid flow driven by capillary action (Figure 3). Figure 3b shows the geometry and dimensions of a microfluidic device. The microfluidic devices were fabricated utilizing a DLP-based 3D printer and using various lamination directions and thicknesses, such as those used for manufacturing the cubic objects. The microchannel features of the 3D-printed microfluidic devices for different lamination directions can be found in the Supplementary Materials. To observe capillary flows inside the microfluidic devices manufactured under different printing conditions, a droplet of 25 μL, consisting of a solution containing DI water and Tween 20 (1 mM), was initially deposited onto a reservoir of the microfluidic device using a micropipette. Subsequently, the displacement of liquid flow driven by capillary action was consecutively recorded using a DSLR camera (EOS 90D, Canon, Japan) equipped with a lens (AF-S NIKKOR 24–70 mm, Nikon, Japan). Finally, the captured images were subjected to digital image processing, using custom MATLAB^®^ R2022a code to obtain data on the temporal progression of liquid flow. All experiments were completed with a minimum of three repetitions, and the reported data represent the average and standard deviations of the results.

## 3. Results and Discussion

**Figure 4:**
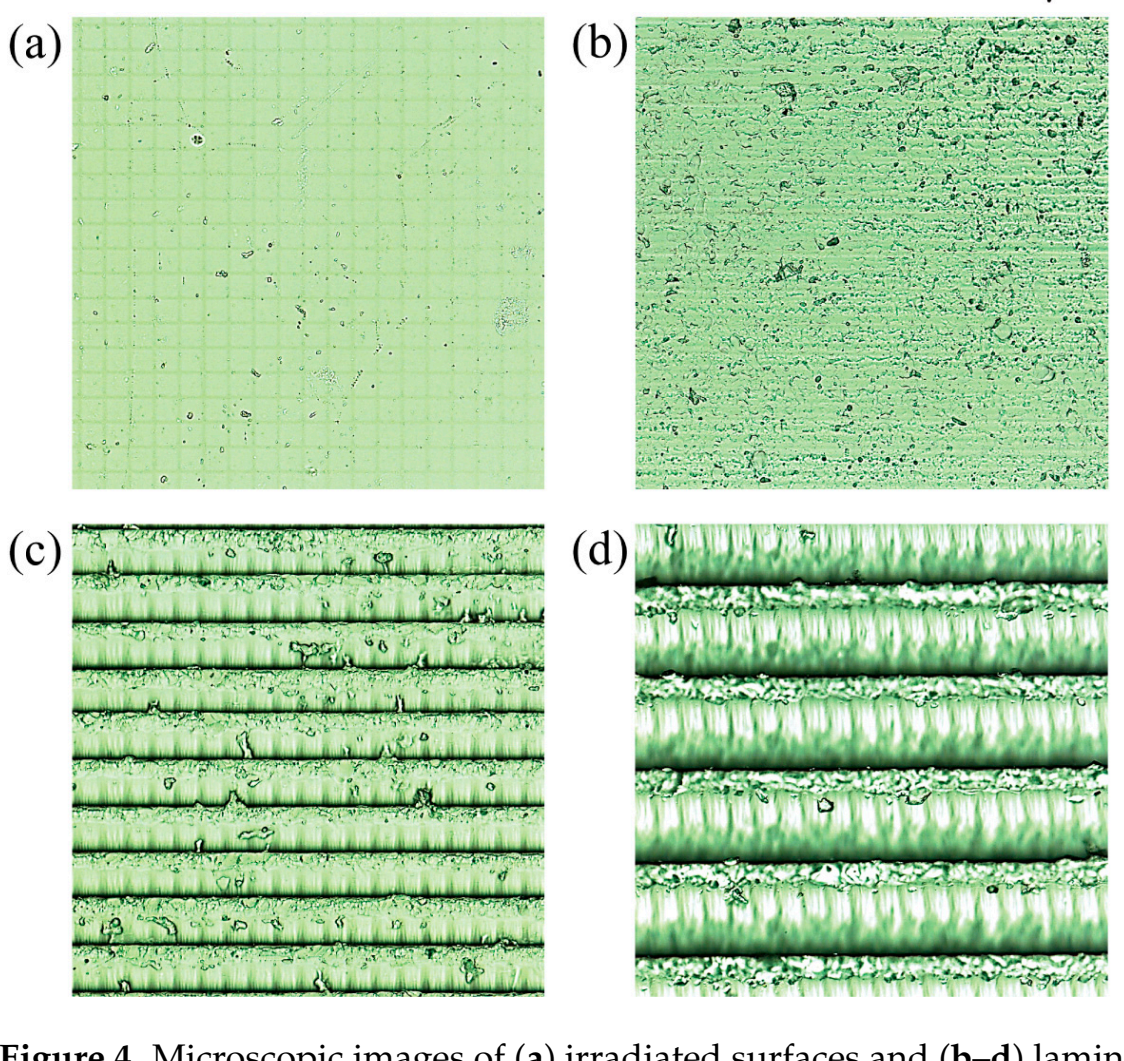
Microscopic images of (**a**) irradiated surfaces and (**b**–**d**) laminated surfaces of 3D-printed objects created under different printing conditions, specifically, horizontal lamination with layer thicknesses of 10 μm, 50 μm, and 100 μm, respectively.

We initially examined the microscopic images of the surfaces of cubic objects created under various printing conditions to qualitatively investigate the relationship between printing conditions and surface roughness (Figure 4). The irradiated surface exhibits the lowest surface roughness compared to the other three surfaces while displaying a microscale lattice pattern of 27 μm in both width and height. This pattern results from the digital micromirror device (DMD) component, which comprises closely grouped small mirrors in a DLP printer. Each mirror of the DMD corresponds to a single pixel, resulting in a lattice pattern that matches the pixel resolution (27 μm) of the DLP printer used in this study. As expected, the surface roughness of the laminated surfaces was observed to increase as the layer thickness increased. In addition, when a layer was cured, the spot closer to the UV light source cured over a larger area than a spot that was farther away. Thus, even in the same layer, a spot closer to the light source had a greater height than other spots.

**Figure 5:**
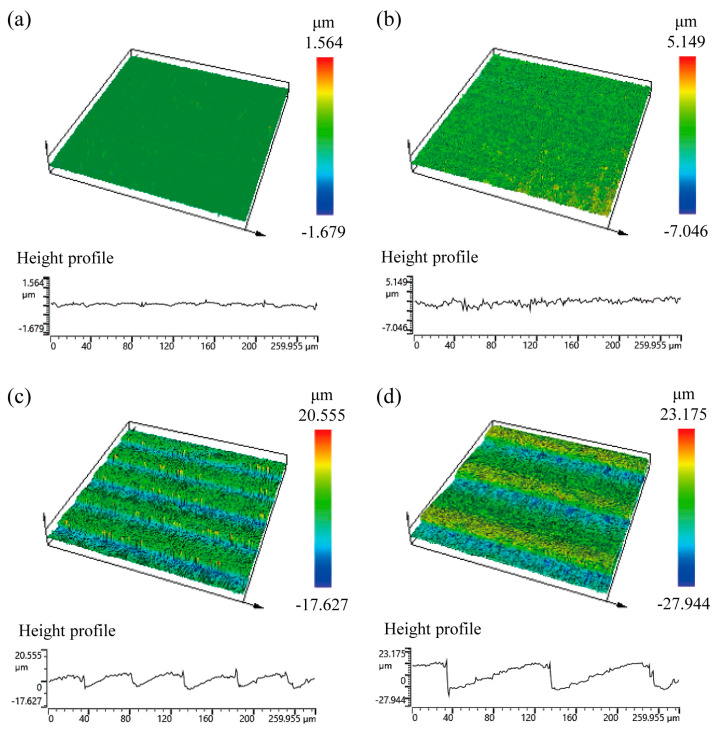
Confocal microscopic images and height profiles of (**a**) irradiated surfaces and (**b**–**d**) laminated surfaces of 3D-printed objects created under different printing conditions, specifically, horizontal lamination with layer thicknesses of 10 μm, 50 μm, and 100 μm, respectively.

We also conducted a quantitative assessment of the surface roughness of 3D-printed objects created under different printing conditions, using a confocal laser scanning microscope (LEXT OLS5000, Olympus, Japan), as shown in Figure 5. Confocal laser scanning microscopy is an optical imaging technique that improves the optical resolution and contrast of a micrograph and has the ability to capture multiple two-dimensional images at different depths within a sample, enabling the reconstruction of three-dimensional structures [37,38]. We employed the arithmetic mean roughness value (*R_a_*) as the representative measure of surface roughness in this case, which was calculated by taking the average of all the profile values in the roughness profile. The arithmetic mean roughness values for irradiated and laminated surfaces with layer thicknesses of 10, 50, and 100 μm were approximately 0.06, 0.38, 2.57, and 7.03 μm, respectively. We quantitatively demonstrated a direct correlation between the increase in layer thickness and the increase in surface roughness. Due to the variations in roughness caused by different layer thicknesses, it is expected that the printing condition of layer thickness can affect surface wettability.

**Figure 6:**
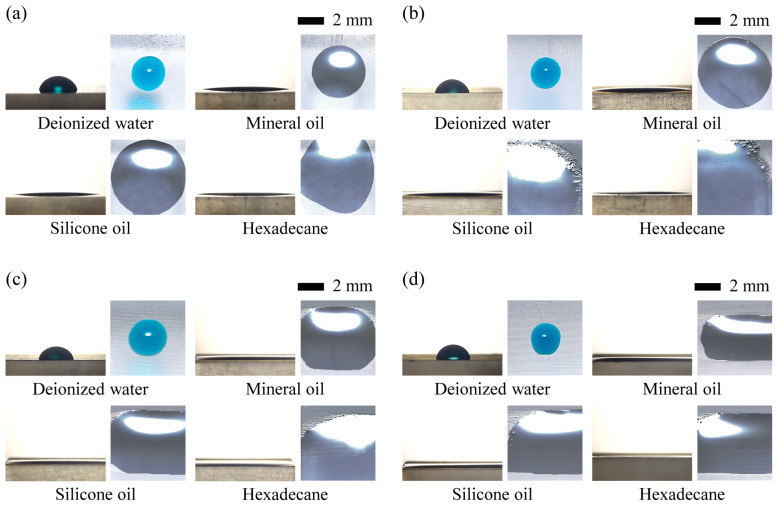
Side and top views of aqueous and oil droplets on: (**a**) irradiated surfaces; and (**b**−**d**) laminated surfaces with layer thicknesses of 10 μm, 50 μm, and 100 μm, respectively.

Figure 6 shows the side and top views of aqueous and oil droplets resting on the irradiated and laminated surfaces of 3D-printed objects created with different layer thicknesses. All liquids, both aqueous and oily, except hexadecane, spread out axisymmetrically on an irradiated surface, giving each droplet the appearance of a sessile droplet. The aqueous droplets on the irradiated and laminated surfaces have an almost sessile droplet shape. However, the droplet shape is slightly distorted by the laminated grain as the layer thickness increases. In the case of oily liquids, the droplets spread unevenly, except on a few surfaces, due to the low surface tension. Based on these findings, we can infer that the thickness or direction of the lamination may affect the fluid dynamics seen on the 3D-printed surface. We will further investigate this assumption through a practical demonstration in the final examination of this study.

**Figure 7:**
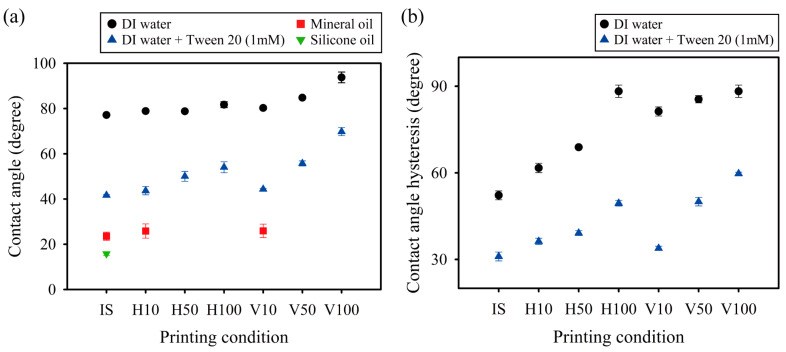
Aqueous and oil droplets on irradiated and laminated surfaces of varying directions and thicknesses: (**a**) contact angle and (**b**) contact angle hysteresis. IS, H, and V stand for irradiated, horizontally, and vertically laminated surfaces, respectively, while the numerical values indicate the lamination thickness.

From the acquired image information in Figure 6, we measured the CAs of water and oil droplets on irradiated and laminated surfaces of different directions and thicknesses (Figure 7a). The CAs of DI water droplets on the horizontally laminated surfaces remained almost constant, regardless of the lamination thickness. However, the CA on the vertically laminated surfaces increased with increasing lamination thickness. The surface, especially at a lamination thickness of 100 μm, showed hydrophobic properties, as indicated by a CA of approximately 94°. This result may have occurred because when DI water droplets are placed on vertically laminated surfaces, they encounter greater resistance and pinning effects from the vertically laminated grain, which prevents them from spreading. As a result, the CA of these droplets is greater than that of droplets placed on horizontally laminated surfaces. However, in the case of droplets containing a mixture of DI water and Tween 20, the CA increases as the thickness increases, regardless of the lamination direction. The disparity in CA tendency between the two aqueous droplets may arise from the addition of surfactant, which reduces surface tension and significantly influences the solid–liquid interaction. Moreover, the CA is significantly greater on vertically laminated surfaces than on horizontally laminated surfaces. As a result, the fluidic behaviors in a 3D-printed microfluidic system are expected to be influenced by the lamination orientation. This will be demonstrated through a final assessment of this work. Finally, the oil droplets are unevenly spread in mineral oil placed on laminated surfaces with thicknesses of 50 and 100 μm and in silicone oil placed on laminated surfaces with thicknesses of 10, 50, and 100 μm, as shown in the top-view images in Figure 6. We were unable to collect any specific data on these behaviors due to the difficulty of determining and precisely measuring the oil droplet contact angle.

The dependence of CAH of aqueous droplets on printing conditions was further investigated, as shown in Figure 7b. In contrast to the CA, it is evident that the lamination direction and thickness influenced the CAH of both aqueous droplets. This difference may be attributed to the fact that CAH is more affected by changes in surface roughness under different printing conditions than CA. Furthermore, the CAH increases with layer thickness, regardless of the lamination direction.

**Figure 8:**
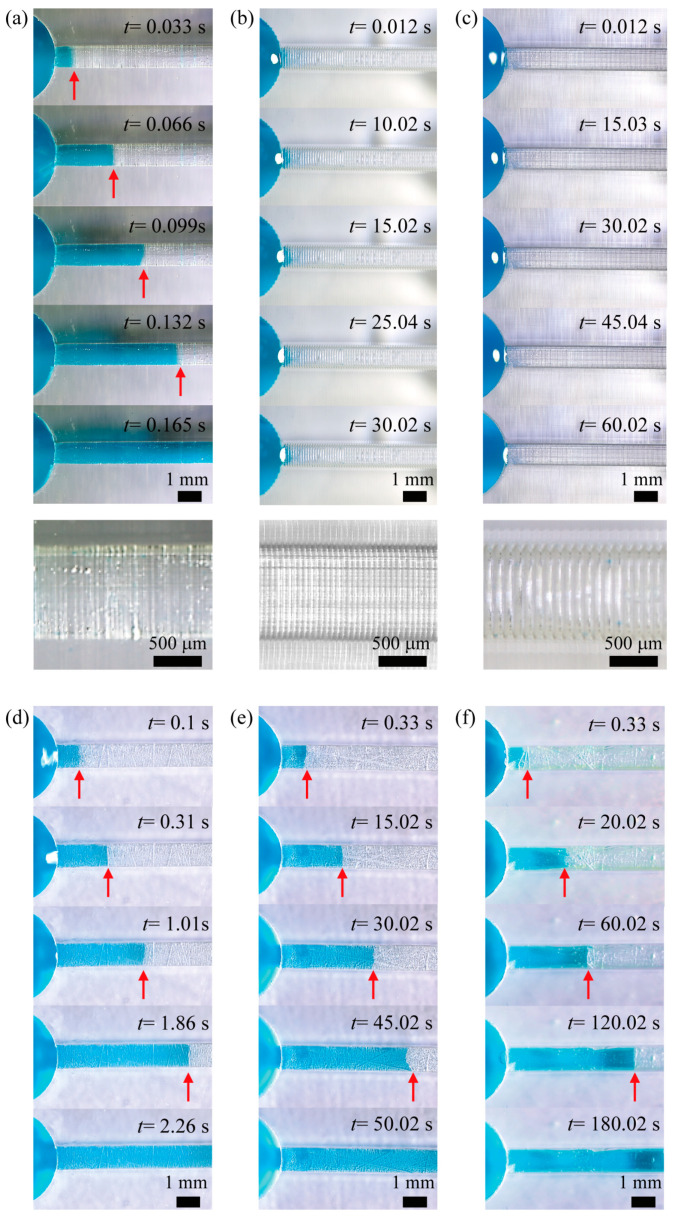
Consecutive images of capillary flow inside microfluidic devices fabricated under different printing conditions: images (**a**–**c**) correspond to printing conditions with a vertical lamination direction and layer thicknesses of 10 μm, 50 μm, and 100 μm, respectively. The bottom row exhibits microscopic photographs of the walls of microchannels created under different printing conditions; images (**d**–**f**) correspond to printing conditions with a horizontal direction and layer thicknesses of 10 μm, 50 μm, and 100 μm, respectively.

To assess the influence of printing conditions on the performance of a DLP 3D-printed microfluidic device, we observed the flow of a liquid consisting of a mixture of DI water and Tween 20 (1 mM), which was propelled by capillary action inside basic microfluidic devices under various printing conditions (Figure 8). A mixture of DI water and Tween 20 was used as the working fluid for the following reasons. The addition of Tween 20 surfactant resulted in a significant decrease in both CA and CAH, as previously shown in Figure 7b. A droplet consisting of a mixture of DI water and Tween 20 showed higher hydrophilic wettability on different printed surfaces than a droplet containing only DI water. In addition, a reduced CAH indicates a reduced pinning force, which hinders the movement of the liquid on the solid surface. As a result, we could experimentally investigate the effect of printing conditions on liquid flow in microchannels to improve capillary action by reducing the liquid surface tension and CAH. In a microfluidic channel created by vertical lamination with a thickness of 10 μm, the liquid rapidly enters the channel. However, when the layer thickness is increased to both 50 μm and 100 μm, the liquid barely flows. Conversely, in a microfluidic channel created via horizontal lamination, the liquid flow slows down as the layer thickness increases. The trends regarding whether the liquid flows and its speed may be correlated with its wetting characteristics, as shown in Figure 7. This correlation will be quantitatively analyzed and discussed later.

**Figure 9:**
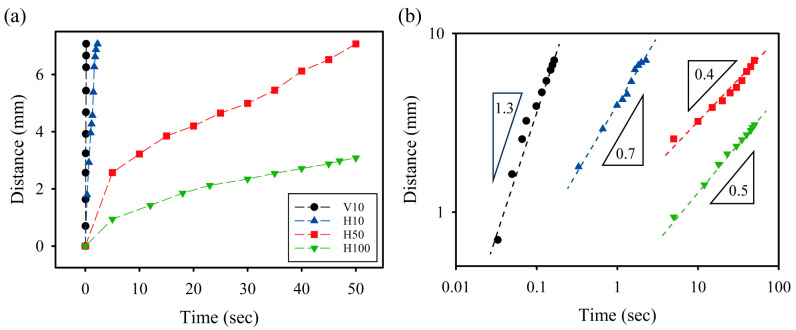
Temporal evolution of capillary flows inside microfluidic devices fabricated under different printing conditions: (**a**,**b**) are represented on a graph using linear and logarithmic scales, respectively. The dashed lines of (**b**) illustrate the power–law correlations between the moving distance of capillary flow and time. The triangle insets show the power–law exponents.

Using the acquired image data from Figure 8, we obtained quantitative information on the temporal evolution of capillary flows in microfluidic devices under different printing conditions (Figure 9). The microfluidic channel created by vertical lamination with a thickness of 10 μm showed the fastest capillary flow. In contrast, the microfluidic channel created by horizontal lamination exhibited slower capillary flow as the thickness increased. Based on the results presented in Figure 7, it is evident that the CA and CAH of droplets containing a mixture of DI water and Tween 20 decrease with decreasing layer thickness. Furthermore, an examination of Figure 7 and Figure 8 shows that the CAH has a more pronounced effect on capillary flow in microfluidic devices than the CA. Contrary to our expectations, the capillary flow in the vertically laminated microchannels was faster than in the horizontal ones with a layer thickness of 10 μm. Possible reasons for this are the uncontrollable scratches found in the horizontally laminated channels and the small CAH in vertically laminated channels. 

By adjusting the linear scales of the *x* and *y* axes in Figure 9a to a logarithmic scale, we established empirical relationships in the form of a power law between the distance traveled and the time needed for capillary flow (Figure 9b). In the case of relatively high capillary flow velocity—that is, in microfluidic channels constructed by laminating vertically and horizontally with a thickness of 10 μm—the distance traveled by the capillary flow tends to be roughly proportional to time. Conversely, in the case of relatively low capillary flow velocity—that is, microfluidic channels constructed by laminating horizontally with a thickness of 50 μm and 100 μm—the distance traveled by the capillary flow tends to be roughly proportional to the square root of time. These tendencies align closely with the power law correlation between the position of the advancing contact line and time, as discussed in previous literature on the dynamics of capillary flows in microchannels [39,40,41,42]. The position of the advancing contact line is known to be proportional to the time when the imbibed liquid passes through the inertial regime at short durations (<1 s, depending on the specific system) [42]. Conversely, the position of the advancing contact line is known to be the square root of time when long durations (<10 s, depending on the specific system) are reached, at which point the liquid enters the viscous or Lucas–Washburn regime [39,40].

The thickness of the horizontally laminated layers in 3D-printed objects has an effect on the surface roughness that can be evaluated by quantitative assessment with a confocal laser scanning microscope, as previously shown in Figure 5. It was also observed that the wetting properties, such as CA and CAH, of droplets containing a mixture of DI water and Tween 20 increased with increasing layer thickness, as previously shown in Figure 7. The wetting properties, especially the CAH, influenced the flow of fluids by capillary action in the 3D-printed microchannels, based on the data in Figure 7 and Figure 9. These results highlight the significance of assessing printing conditions before carrying out practical research when utilizing DLP printers to produce microfluidic devices. 

## 4. Conclusions

This study investigates the surface wetting properties (e.g., CA and CAH) of 3D-printed items under different DLP printing conditions, including lamination direction and thickness. The thickness of the horizontally laminated layers in 3D-printed objects had an effect on the surface roughness, as seen in quantitative assessment with a confocal laser scanning microscope. The CA of the DI water droplets remained nearly constant on horizontally laminated surfaces but increased with increasing thickness on vertically laminated surfaces. Regardless of lamination direction, the CA increased with thickness for droplets containing a mixture of DI water and Tween 20. In contrast, the lamination direction and thickness influenced the CAH of both types of aqueous droplets. Moreover, the CA and CAH for oil droplets were limited to specific surfaces, due to uneven spreading. Thus, printing conditions showed a noteworthy effect on the efficiency of a microfluidic device produced through DLP 3D printing, as evidenced by the observed flow of liquid through microfluidic channels, driven by capillary action. This study offers essential and vital insights into grafting DLP-based 3D printing in various microfluidic applications, such as chemistry, materials science, medicine, biology, pharmaceuticals, and healthcare. 

## Figures and Tables

**Table 1:** Physical properties of the aqueous and oil–liquid phases (23 ± 1 °C).

	Density (kg/m^3^)	Viscosity (mPa·s)	Surface Tension (mN/m)
DI water	998	0.93	71.8
DI water + Tween 20 (1 mM)	998	1.01	37.5
Mineral oil	830	9.13	37.6
Silicone oil, 50 cSt	960	48	36.9
Hexadecane	774	3.46	27.3

## Data Availability

Data are contained within the article and supplementary materials.

## Supplementary Materials

The following supporting information can be downloaded at: https://www.mdpi.com/article/10.3390/mi15010061/s1, Figure S1: Schematic diagrams of (a) the microfluidic channels with vertical lamination and (b) the microfluidic channels with horizontal lamination.
