# Supplementary material for: Surface-Wetting Characteristics of DLP-Based 3D Printing Outcomes under Various Printing Conditions for Microfluidic Device Fabrication
Source: Micromachines (Basel). 2023 Dec 28;15(1):61. doi: 10.3390/mi15010061 (PMC10820386; doi:10.3390/mi15010061)
Supplement: Supplementary file 1 [file micromachines-15-00061-s001.zip › micromachines-2747326-supplementary.pdf]

Supplementary Material

Surface-Wetting Characteristics of DLP-Based 3D Printing Outcomes under Various Printing Conditions for Microfluidic Device Fabrication

Jeon-Woong Kang, Jinpyo Jeon, Jun-Young Lee, Jun-Hyeong Jeon and Jiwoo Hong *

School of Mechanical Engineering, Soongsil University, 369 Sangdo-ro, Dongjak-gu, Seoul 06978, Republic of Korea; kangjw159@gmail.com (J.-W.K.); junjynpyo@gmail.com (J.J.); ktino27@gmail.com (J.-Y.L.); bread951030@gmail.com (J.-H.J.)

* Correspondence: jiwoohong@ssu.ac.kr

3D-Printed Microchannels Features for Different Lamination Directions

In the case of a microchannel created by printing with vertical lamination, all planes consist of vertically laminated surfaces, as shown in Figure S1a. In the case of microchannels created by printing with horizontal lamination, the sides of the channel have horizontal laminated surfaces, but the bottom has an irradiated surface, as shown in Figure S1b. Consequently, it is unable to form a microchannel in which every plane is made up of irradiated surfaces.

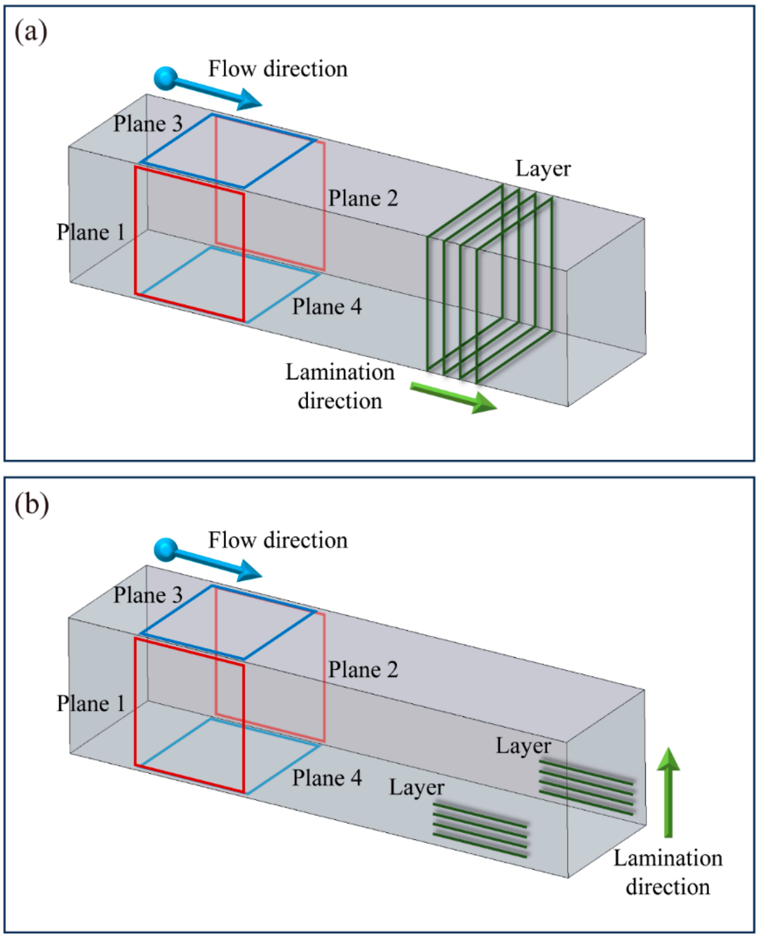

Figure S1. Schematic diagrams of (**a**) the microfluidic channels with vertical lamination and (**b**) the microfluidic channels with horizontal lamination.
